# Supplementary material for: Virus Induced Lymphocytes (VIL) as a novel viral antigen-specific T cell therapy for COVID-19 and potential future pandemics
Source: Sci Rep. 2021 Jul 27;11:15295. doi: 10.1038/s41598-021-94654-y (PMC8316478; doi:10.1038/s41598-021-94654-y)

Supplementary Fig. S1: Expression of cytolytic markers among rapidly-expanded SARS-CoV-2 and CMV antigen-specific VIL. **a**, Representative flow-cytometric analysis showing expression of CD107a, Perforin and Granzyme B among enriched and expanded CMV-specific $CD8^+$ T cells at day-7. **b**, Summary of data obtained in **a**, *(n=3)*. **c**, Analysis as in **a**, for SARS-CoV-2-specific $CD8^+$ T cells, and **d**, analysis as in **b**, for SARS-CoV-2-specific $CD8^+$ T cells *(n=3)*.

Supplementary Fig. S2: Co-expression of immune checkpoint markers among rapidly-expanded SARS-CoV-2 and CMV antigen-specific VIL. **a**, Representative flow-cytometric analysis showing co-expression of PD-1 with TIM-3, LAG-3 or TIGIT among enriched and expanded CMV-specific $CD8^+$ T cells at day-7. **b**, Summary of data obtained in **a**, *(n=3)*. **c**, Analysis as in **a**, for SARS-CoV-2-specific $CD8^+$ T cells, and **d**, analysis as in **b**, for SARS-CoV-2-specific $CD8^+$ T cells *(n=3)*.

Supplementary Figure S1

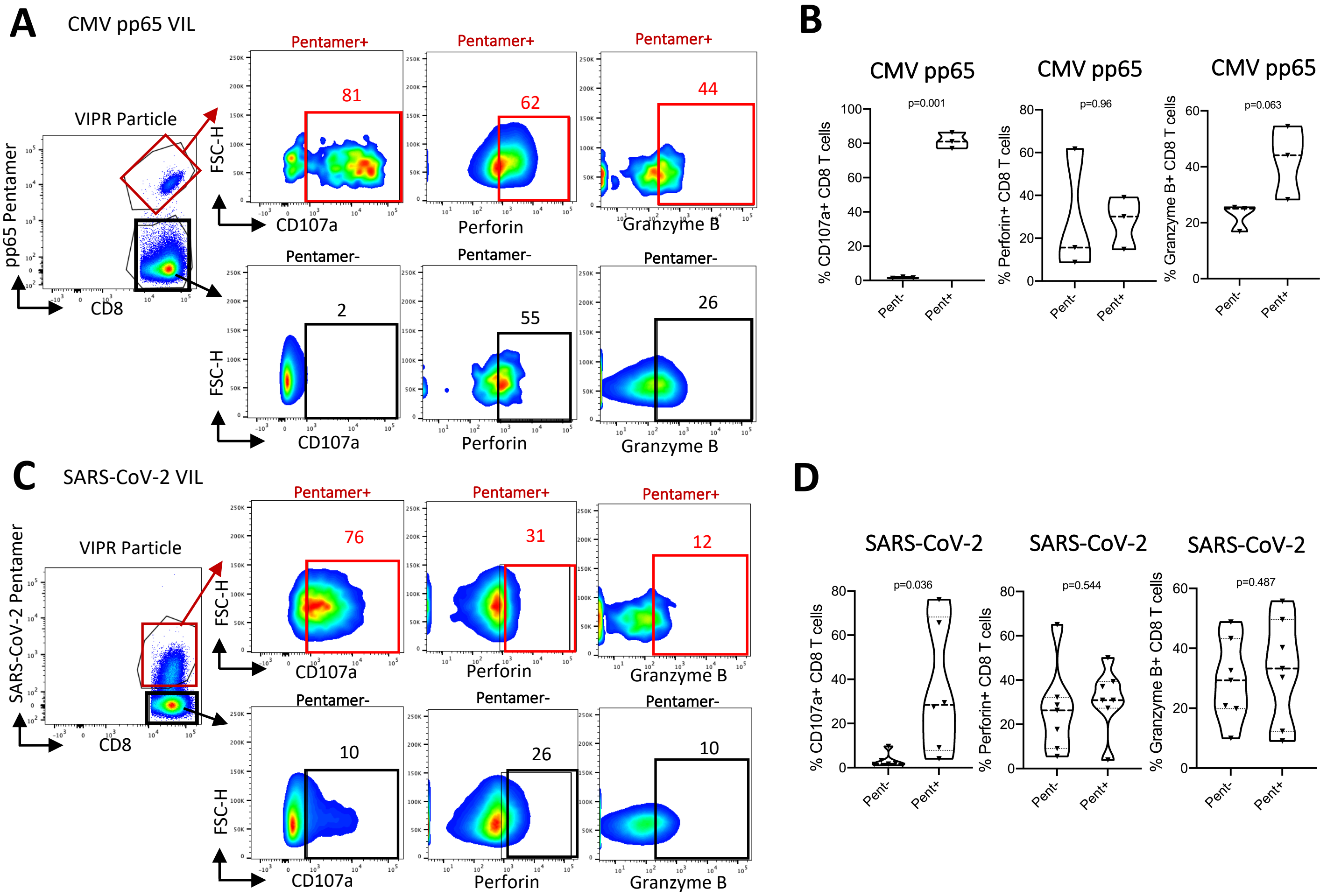

Supplementary Figure S2

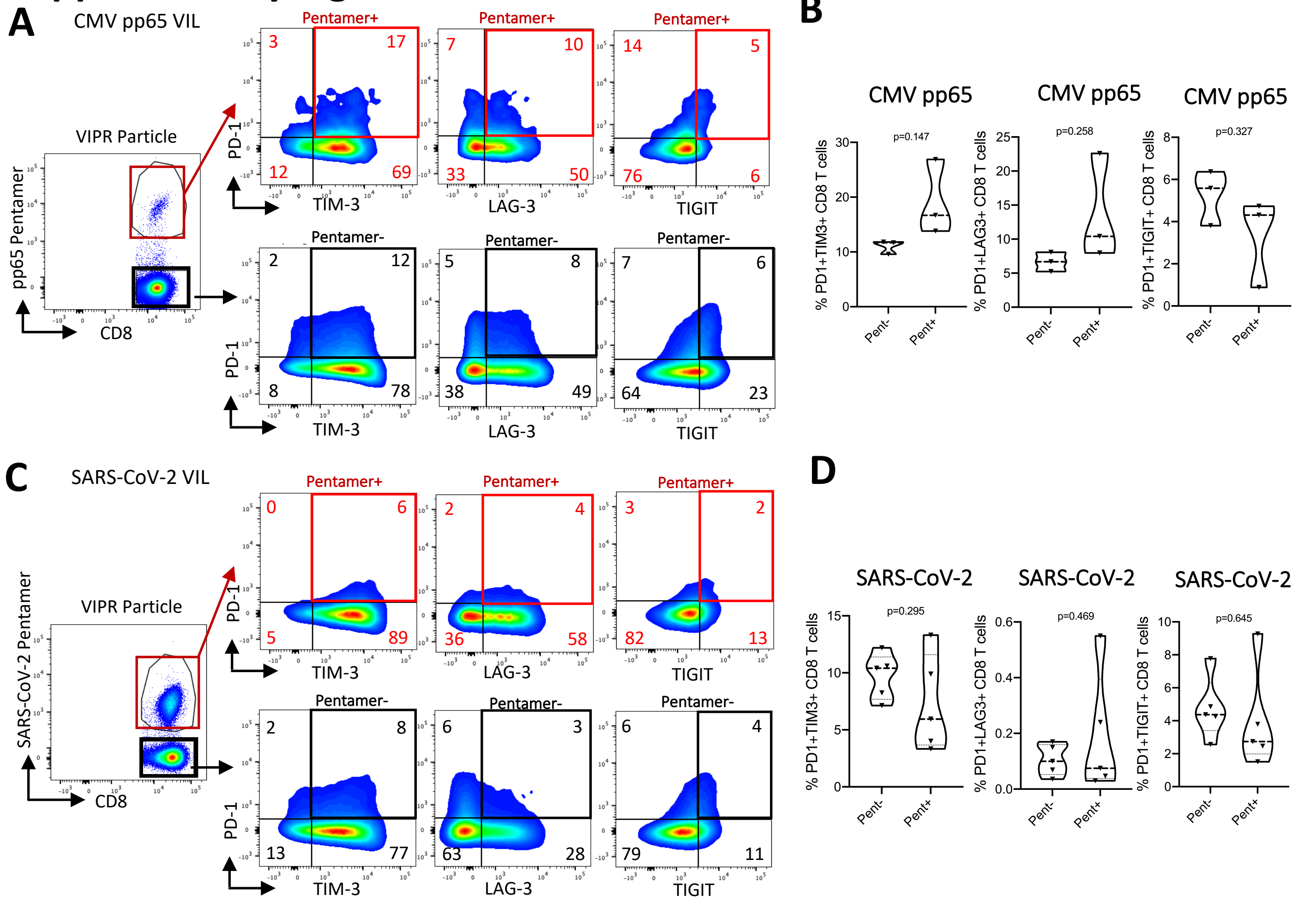

Supplement: Supplementary file 1 — Supplementary Information. [file 41598_2021_94654_MOESM1_ESM.pdf]
